# Genome-wide identification of the WRKY transcription factors family and regulation of metabolites under cold stress in *Astragalus membranaceus*

**DOI:** 10.1186/s12870-025-07685-2

**Published:** 2025-11-28

**Authors:** Keyong Zhang, Jiajia Chen, Jicheng Liu, Hui Li, Ming Jiang

**Affiliations:** 1 Qiqihar Medical University, Qiqihar, Heilongjiang 161006 China; 2 Heilongjiang Provincial Key Laboratory of Research and Utilization of Distinctive Northern Medicinal Resources, Qiqihar, Heilongjiang 161006 China

**Keywords:** WRKYs Family, *Astragalus Membranaceus*, Cold Stress, Metabolic Profiling

## Abstract

**Background:**

WRKY transcription factors (TFs) are important transcriptional regulators in plants, with their members widely involved in plant growth and development as well as responses to abiotic stresses. However, researches on WRKY genes in the medicinal plant *A. membranaceus* are scarce. Specifically, the roles of *AmWRKYs* in cold stress adaptation and their regulatory effects on flavonoid biosynthesis, which determines both medicinal quality and stress resistance, remain largely unexplored. Given its high economic value and extreme sensitivity to cold in its main cultivation regions, identifying key regulators of its cold tolerance is crucial for genetic improvement.

**Result:**

In this study, 94 *AmWRKY* were identified based on genome analysis, distributed across 8 chromosomes. *AmWRKYs* are structurally conserved, all carrying the core conserved domain "WRKYGQK" and classified into 6 subgroups. Cis-acting elements responsive to plant growth and development, abiotic stress, and hormone responses were identified in the promoter regions. Additionally, the transcriptome and metabolome data under cold stress were analyzed, and a co-expression and metabolite association network of *AmWRKY* genes was constructed. Sixteen *AmWRKY* transcription factors showed dynamic expression under cold stress, among which *AmWRKY22/24/44/65* were continuously upregulated, indicating their core roles in cold adaptation. Co-expression network analysis revealed the synergistic effects of *AmWRKY* with AP2/ERF, MYB, and NAC transcription factors, forming a regulatory module integrating hormone signaling, antioxidant pathways, and circadian rhythm regulation. Metabolomics analysis indicated that *AmWRKY24/44* expression was positively correlated with the upregulation of key flavonoid biosynthesis genes (*AmCHS*, *AmFLS*) and the accumulation of nine cold-responsive flavonoids. These findings suggest a new regulatory pathway of *AmWRKY24/44* → flavonoid biosynthesis → cold resistance, linking secondary metabolism with environmental adaptation.

**Conclusion:**

This study reveals a novel regulatory pathway—“*AmWRKY24/44*” → flavonoid biosynthesis → cold resistance—in *A. membranaceus*, providing deeper mechanistic insights into how WRKY transcription factors modulate secondary metabolism under cold stress. These findings offer a valuable theoretical foundation for genetic improvement of cold tolerance in this medicinally important species.

**Supplementary Information:**

The online version contains supplementary material available at 10.1186/s12870-025-07685-2.

## Introduction

*Astragalus membranaceus* (Fisch.) Bunge (*A. membranaceus*) is an important medicinal plant with a history of over 2000 years of application [1]. Modern research indicates that it has remarkable effects in the prevention and treatment of hepatitis, diabetes, coronary heart disease, bronchitis, and viral myocarditis [2]. The main active ingredients that exert pharmacological effects include flavonoids, saponins and polysaccharides, among which flavonoids exhibit significant anti-tumor, antioxidant and anti-inflammatory activities [3, 4]. The National Health Commission of China has included *A. membranaceus* of Food and Medicine Homology. The Heilongjiang Province has formulated local food standards for Dried Astragalus Flower and Dried Astragalus Stem and Leaf, which has greatly enhanced the development value of *A. membranaceus* in the fields of medicine and food. With the modernization of traditional Chinese medicine and the increasing global demand for health, *A. membranaceus* has seen a continuous rise in market demand.

The main production areas of *A. membranaceus* are located in the high-altitude and cold regions of northern China, where it has a long growth cycle and is extremely sensitive to cold [5]. Extreme climates such as spring cold, late spring cold and early frost often cause damage to the young seedlings, poor root development and even large-scale death, resulting in a 30% to 50% reduction in medicinal materials production [6]. This seriously threatens the economic benefits of the production areas and the safety of the Chinese medicinal materials supply. Therefore, elucidating the molecular mechanism of *A. membranaceus* in resisting cold stress and breeding cold-resistant varieties have become an urgent need to ensure the sustainable utilization of Chinese medicinal materials resources.

Plants respond to cold stress in a complex manner from multiple aspects, including endogenous hormones, membrane composition, antioxidant components, gene transcription levels, and specific protein expression. Research shows that cold stress promotes the accumulation of secondary metabolites in plants, such as terpenoids, alkaloids, and flavonoids, especially in medicinal plants where the accumulation of secondary metabolites is more pronounced [7]. Flavonoids, as crucial secondary metabolites, help plants cope with abiotic stresses such as cold by increasing in concentration to mitigate free radical damage [8]. Specifically, when plants are exposed to cold, flavonoids accumulate in the lipid phase of the cell membrane, thereby maintaining the stability of the cell membrane [9]. Presently, related research predominantly centers around aspects such as the agronomic characteristics, cultivation techniques, development and utilization of *A. membranaceus*, as well as the extraction of its active components and functional evaluation. Nevertheless, investigations into the molecular mechanism underlying its resistance to cold stress, the biosynthetic pathways of flavonoids, and the functional analysis of key enzyme genes remain relatively scarce.

As a crucial family of transcriptional regulators, WRKY proteins modulate diverse processes by binding to the W-box cis-element (TGACC(A/T)) in target gene promoters, forming complex regulatory networks [10, 11]. While the evolutionary history of this family has been characterized by expansion through various duplication events in species like *Solanum* and soybean [12, 13], and members play roles in development and immunity [14–16]. Their defined function in mediating cold stress responses, particularly through the regulation of protective secondary metabolites like flavonoids, which is of paramount relevance to this study.

The pivotal regulatory function of WRKYs in cold stress responses has been systematically validated through multi-species experimental systems. WRKYs demonstrates marked upregulation during early cold stress phases in white clover, indicating its involvement in rapid cold signal transduction initiation [17]. *Cenchrus purpureus* studies reveal distinct cold expression patterns of specific WRKY members, whose overexpression significantly enhances cold tolerance, confirming functional conservation across species [18]. *Forsythia suspensa* research demonstrates WRKY dynamic expression regulation throughout cold adaptation processes from seed germination to fruit ripening, with characteristic expression profiles underscoring its critical role in environmental adaptability [19]. *Platycodon grandiflorus* investigations further uncover temperature-responsive specificity, suggesting certain members may establish cold tolerance regulatory nodes through preferential expression patterns [20]. Experiments provide direct phenotypic evidence, showing significant positive correlations between elevated WRKY expression and cold-tolerant phenotypes under cold stress in *Ginkgo biloba* [21]. *Lagerstroemia indica* studies reveal substantial WRKY expression reprogramming during cold stress, implicating its regulatory role in cold adaptation through downstream gene modulation [22]. These findings collectively elucidate WRKY multi-tiered regulatory mechanisms under cold stress, spanning molecular signaling, functional verification, and adaptive regulation. However, systematic research on *AmWRKY* under cold stress is still lacking.

Based on the available genomic data of *A. membranaceus*, we identified *AmWRKY* and conducted a detailed analysis of its chromosomal location, gene structure, phylogenetic relationship and cis-acting elements. Additionally, through the correlation analysis of transcriptome and metabolome data under cold stress, the gene expression pattern, co-expression network and regulation of metabolite accumulation of *AmWRKY* were revealed. These results are of great significance for analyzing the molecular response mechanism under cold stress and also provide valuable information for the identification and application of *AmWRKY*.

## Materials and methods

### Plant materials and growth environment

The seeds of *A. membranaceus* used in the experiment were collected from the Greater Khingan Mountains in Heilongjiang Province and authenticated by Associate Professor Hui Li from Qiqihar Medical University. The seeds were sown in trays, then cultivated in a light incubator. After germination, the seedlings were transplanted into seedling cups and placed in an artificial climate chamber for further cultivation. The conditions were as follows, a 14-h light period at 24 °C, a 10-h dark period at 20 °C, a humidity of 55%, and a light intensity of 10,000 lx. After continuous cultivation for 48 days, the seedlings were subjected to cold stress treatment. The cold treatment was set at 4 °C, with a 14-h light period and a 10-h dark period (day/night), a humidity of 55%, and a light intensity of 10,000 lx. The cold treatment lasted for 24 h. Three groups included the control group (CK), the 12-h cold treatment group (Cold12h), and the 24-h cold treatment group (Cold24h) were set up. Leaf samples were randomly collected from the seedlings (20 seedlings as one biological replicate, with three replicates per group). The collected leaves were cut into small pieces, mixed evenly, immediately cooled in liquid nitrogen, and stored at −80 °C for subsequent research.

### Genome-wide identification of *AmWRKY*

The *A. membranaceus* genome-wide data and annotation files used in this study were obtained from the NCBI Genome Database (accession number: GCA_039519185.1). The corresponding data of the model plant *Arabidopsis thaliana* were retrieved from the Ensembl Plants database (https://plants.ensembl.org/index.html), and the WRKY protein sequences were sourced from the NCBI database. The genome-wide protein sequences of *A. membranaceus* were extracted using TBtools (v2.025), and the initial screening was conducted using the Hidden Markov Model (HMMER v3.3.2) based on the WRKY domain feature model (PF03106) from the Pfam database (v35.0). To ensure domain integrity, all candidate protein sequences were subsequently verified using the NCBI Conserved Domain Database (CDD) and manual inspection to confirm the presence of the complete 'WRKYGQK' heptapeptide motif and the associated zinc-finger structure. To eliminate redundancy from alternative splicing variants or highly identical paralogs, a phylogenetic redundancy check was performed. Multiple sequence alignment was conducted using ClustalW in MEGA, and sequences exhibiting 100% identity in the WRKY domain and > 95% full-length sequence similarity were considered redundant. For each redundant set, only the longest transcript isoform was retained for further analysis. The physicochemical properties of *AmWRKY* proteins were systematically analyzed using the Protein Parameter Calc module of TBtools, including key parameters such as amino acid number (AA), molecular weight (MW), theoretical isoelectric point (pI), instability index (II), aliphatic index (AI), and average hydrophilicity (GRAVY).

### Chromosome localization and duplication events of *AmWRKY* gene family

Based on the *A. membranaceus* genome annotation file, the chromosome localization information of *AmWRKY* was extracted using the Genome Annotation module of TBtools (v2.025). A high-resolution chromosome distribution map was generated using the Chromosome Map Visualization module (parameters: gene density bin = 100 kb). The potential duplication events of *AmWRKY* gene family were analyzed using the MCScanX software, and the corresponding gene duplication map was drawn using Tbtools.

### Phylogenetic analysis of *AmWRKY*

To explore the evolutionary characteristics of *AmWRKY*, the WRKY protein sequences of *Arabidopsis thaliana* were obtained from the NCBI database and subsequently integrated with *AmWRKY* protein sequences for phylogenetic analysis. The amino acid sequences of WRKY from the two species were aligned using MEGA 11.0 software, and a phylogenetic tree was constructed using the Neighbor-Joining (NJ) method. To assess the reliability of the branches, bootstrap resampling was set to 1000 times, and other parameters were kept as default. Finally, the phylogenetic tree was optimized and visualized through the iTOL online platform (https://itol.embl.de). To study the potential evolutionary mechanism of *AmWRKY*, we integrated the genome data of *Arabidopsis thaliana* (TAIR10), *Glycine max* (NCBI Assembly GCF_000004515.6) and *A. membranaceus*. Whole-genome synteny analysis was then conducted using MCScanX (v1.1). The cross-species synteny blocks were visualized using the Advanced Synteny Analysis module of TBtools, and the WRKY homologous gene pairs with evolutionary conservation were screened.

### Analysis of *AmWRKY* gene structure, motifs and cis-acting elements

To systematically analyze the structural characteristics and functional conservation of *AmWRKY*, the untranslated regions (UTRs), coding sequences (CDSs), and intron–exon distributions were visually analyzed. A maximum likelihood phylogenetic tree was constructed through the One Step Build a MLtree module. Further, the MEME online tool (parameters set: number of motifs = 6, others default) was used to mine conserved motifs. Multiple sequence alignment (ClustalW algorithm) was completed using MEGA 12 software, and the results were visualized after filtering out redundant sequences. The 2000 bp upstream sequence was selected as the promoter region, and cis-acting elements were predicted using the PlantCare website (http://www.plantcare.co.uk/). The results were visualized using the TBtools software.

### Expression analysis of *AmWRKY* under cold stress and qRT-PCR validation

Based on the RNA-seq data of *A. membranaceus* under cold stress (https://www.cncb.ac.cn/; CRA031815), the FPKM values of *AmWRKY* were extracted. The expression profile was visualized, and all FPKM values were normalized by row. To verify the reliability of the RNA-seq data, qRT-PCR validation was conducted using cDNA from *A. membranaceus* leaf tissues as the template. The relative expression levels of differentially expressed genes (DEGs) in the CK, Cold12h, and Cold24h treatment groups were calculated using the 2^−ΔΔCt^ method based on CT values. Three biological replicates were set for each sample, and each biological replicate was performed three times technically. Total RNA from *A. membranaceus* leaves under cold stress was first extracted using the RNAprep Pure Plant Total RNA Extraction Kit (DP432), and then cDNA templates were synthesized using the TIANScript II cDNA First-Strand Synthesis Kit (KR107). The qRT-PCR experiment was conducted using the FastFire Quick Fluorescence Quantitative PCR Premix (SYBR Green) (FP207) on the ABI QuantStudio6 real-time fluorescence quantitative PCR instrument. The reaction program was set as follows: 1 min at 95 °C for pre-denaturation, followed by 40 cycles (5 s at 95 °C, 15 s at 60 °C). Ten DEGs were selected for qRT-PCR validation analysis. The internal reference gene GAPDH was the reference gene developed in the laboratory for *A. membranaceus*, and the primers were synthesized by Sangon Biotech (Shanghai) Co., Ltd. The primer information is shown in Table S1.

### Non-targeted metabolic analysis of *A. membranaceus* under cold stress

A systematic analysis was carried out based on the metabolic dynamics of *A. membranaceus* at different time points during cold stress (CK, Cold12h, and Cold24h, with three biological replicates set for each group). The non-targeted metabolome detection platform of Metware Metabolic Biotechnology Co., Ltd. (Wuhan) was employed. It was performed using an UHPLC system (Shimadzu Nexera X2) coupled with a tandem mass spectrometer (Applied Biosystems 4500 QTRAP). Chromatographic separation was achieved using an Agilent SB-C18 column (2.1 × 100 mm, 1.8 μm) with a gradient elution program (0.35 mL/min flow rate, 40 °C column temperature) using 0.1% formic acid in water (A) and 0.1% formic acid in acetonitrile (B) as mobile phases with the following gradient: 5% B at 0 min, linearly increased to 95% B at 9.0 min, maintained at 95% B for 1 min, then reduced to 5% B at 11.1 min and equilibrated until 14 min. Data were acquired in both positive and negative ionization modes with electrospray ionization (ESI) to ensure comprehensive metabolite detection. Significantly differential metabolites were screened with criteria of |log₂ fold change (FC)|≥ 1.5 and false discovery rate (FDR) < 0.05 to control for false positives due to multiple testing, metabolic pathway annotation and enrichment analysis were performed in combination with the KEGG database.

### Data statistical analysis

Data statistics were conducted using Excel, and GraphPad Prism (version 9.0) was employed for significance and correlation analysis. One-way ANOVA was used to assess inter-group differences, with Duncan's multiple range test (*p* < 0.05) for significance. Cytoscape (v3.9.1) software was utilized to visualize co-expression network diagrams.

## Results

### Identification and analysis of *AmWRKY* gene family

Following the verification of domain integrity and phylogenetic redundancy elimination screening, a total of *94 AmWRKY* genes were ultimately identified (Table S2). These proteins were designated as *AmWRKY1*-*AmWRKY94* according to the order of their chromosomal localization. Systematic analyses indicated that the lengths of the protein sequences within this family exhibited notable variations, ranging from 60 to 1,625 amino acids (aa). *AmWRKY77* encoded the shortest polypeptide, consisting of 60 aa with a molecular weight (MW) of 7.11 kDa, while *AmWRKY39* represented the largest protein, containing 1,625 aa and having a MW of 185.71 kDa. The theoretical isoelectric points (pI) spanned from 4.87 (*AmWRKY93*) to 10.02 (*AmWRKY77*), suggesting the presence of both acidic (pI < 7) and basic (pI > 7) protein subtypes within this family. Analysis of key physicochemical parameters showed that the instability index (II) values ranged from 25.80 (AmWRKY18) to 70.74 (AmWRKY57). Notably, 85 out of the 94 identified AmWRKY proteins (approximately 90.4%) exhibited an II value greater than 40, confirming that the majority of these proteins are unstable in vitro.

The aliphatic index of the AmWRKY proteins varied from 39.72 (AmWRKY44) to 88.52 (AmWRKY38), with a mean value of 61.55, reflecting a moderate overall abundance of hydrophobic groups. The grand average of hydropathicity (GRAVY) for all 94 members ranged from −1.356 (AmWRKY63) to −0.188 (AmWRKY18), with a mean value of −0.77 ± 0.12 (t-test, *p* < 0.01), thus validating the overall hydrophilic nature of the AmWRKY family. In silico prediction of subcellular localization suggested that AmWRKY proteins are predicted to be extensively distributed within various cellular compartments of *A. membranaceus*. Eighty-three AmWRKY proteins were predicted to localize in the nucleus, 9 in the chloroplast, and 2 in the mitochondria (Table S3; https://wolfpsort.hgc.jp/). The subcellular distribution pattern of AmWRKY proteins, predominantly in the nucleus with involvement in the chloroplast and mitochondria, suggests that these proteins are likely to participate in diverse biological processes.

### Chromosome localization and duplication events analysis of *AmWRKY*

**Fig. 1:**
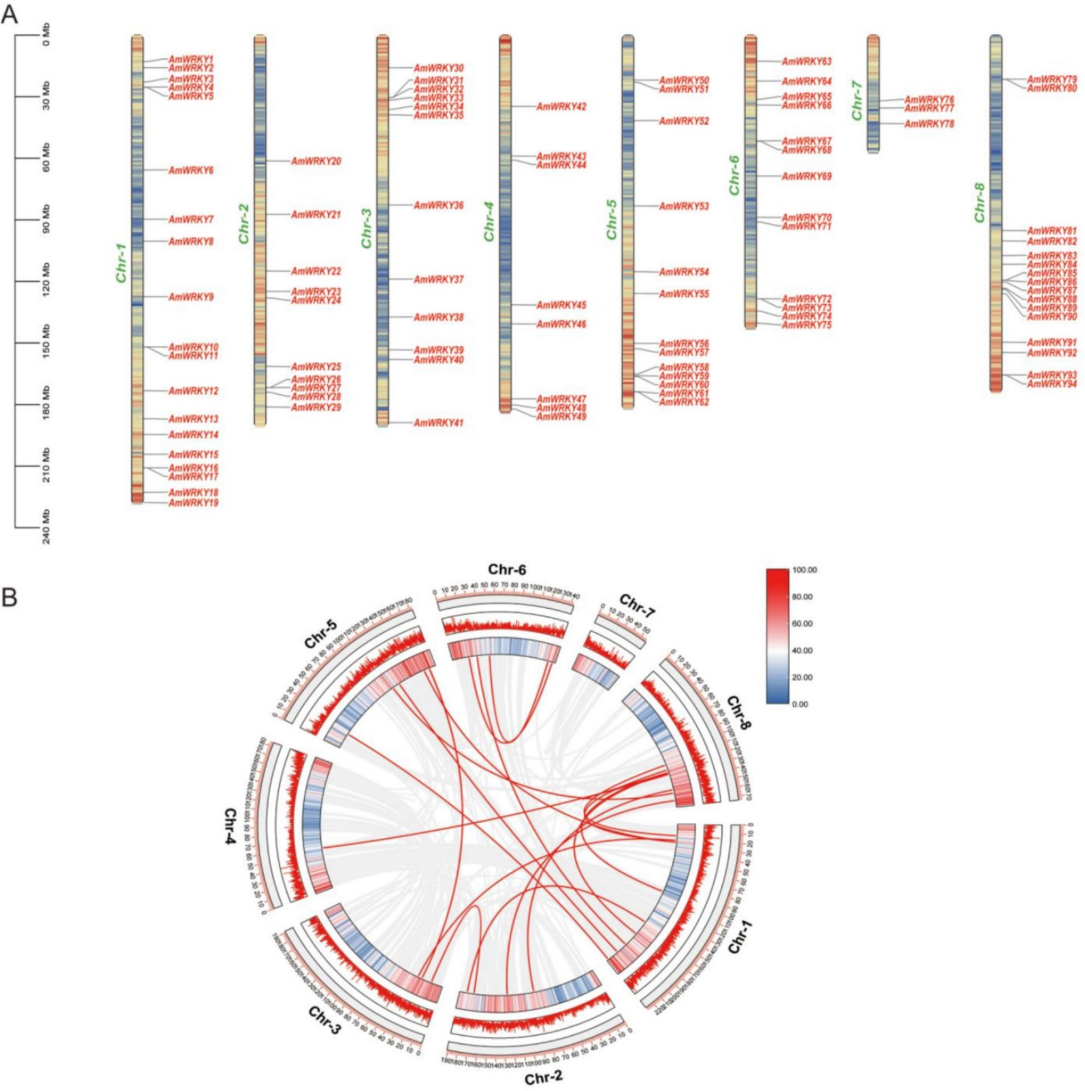
Chromosome localization and gene replication events of *AmWRKY* in *A. membranaceus*. **A** Localization information of 94 *AmWRKY* genes across the eight chromosomes. **B** Gene duplication events of *WRKY* genes in. The grey lines represent all collinearity genes, and the red line represents the tandem replication line relationship between *AmWRKY* genes. The outer two circles represent gene density information, red represents high gene density, whereas blue represents low density

The chromosome distribution pattern of *AmWRKY* was systematically analyzed. A total of 94 *AmWRKY* genes were unevenly distributed across 8 chromosomes (Chr1-Chr8) (Fig. 1A), showing a significant telomere enrichment feature (72 genes were clustered within the 300 Mb regions at both ends of the chromosomes). The distribution density on chromosomes varied significantly. Chr1 carried the most members (19 genes, accounting for 20.2%), while Chr7 contained the fewest (3 genes, accounting for 3.2%), which might be related to the chromosome length (Chr1: 152.7 Mb vs. Chr7: 82.4 Mb) and local recombination rate.

To ensure high-confidence genomic context for the subsequent analysis of gene distribution, duplication, and synteny, we focused exclusively on genes anchored to the eight primary chromosomes. All genes located on unassembled contigs or scaffolds were excluded from this study. The analysis of the duplication events of the *AmWRKY* gene family indicated that a total of 20 pairs of segmental duplicated genes were identified on chromosomes 1, 2, 3, 4, 5, 6, and 8, while no duplicated gene pairs were found on chromosome 7 (Fig. 1B). Ka/Ks analysis was conducted on these duplicated gene pairs to assess the selection pressure (Table S4). We found that the Ka/Ks values of most gene pairs were less than 1, suggesting that these genes tended to maintain sequence stability during evolution. Analysis of duplication events indicated that segmental duplication, rather than tandem duplication, was the primary expansion mechanism for the AmWRKY gene family.

### Phylogenetic analysis and classification of *AmWRKY*

**Fig. 2:**
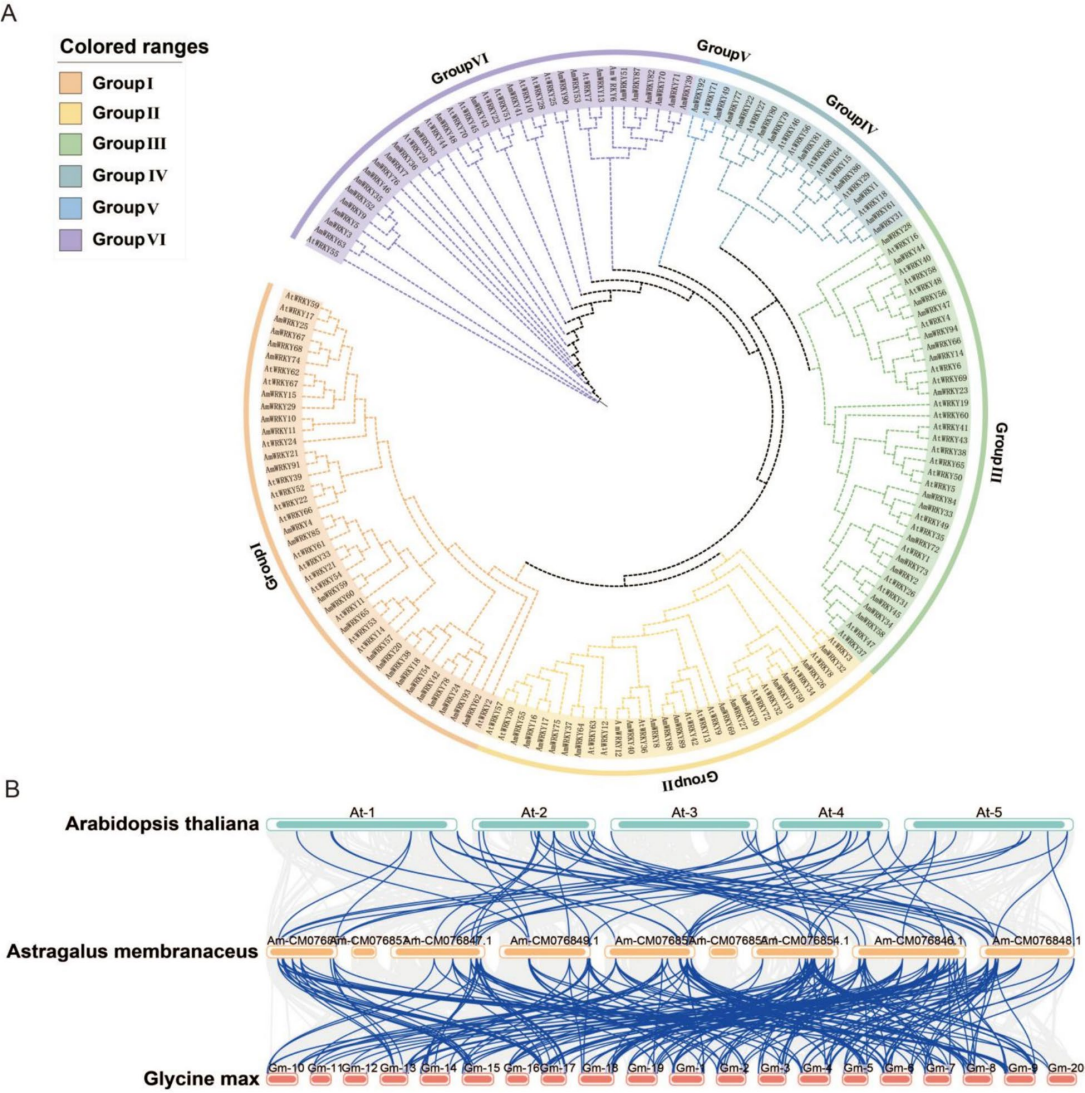
The NJ phylogenetic tree analysis of WRKY*.* **A** The NJ phylogenetic tree constructed from *A. thaliana* and AmWRKY proteins. The bootstrap value is set to 1000 repetitions. Different colors represent different subfamilies. **B** Genome collinearity analysis of *WRKY* genes among *Arabidopsis thaliana* (At), *Astragalus membranaceus* (Am), and *Glycine max* (Gm). Chromosomes of each species are represented as labeled ideograms (e.g., AtChr1, AmChr1, GmChr1). Blue lines indicate syntenic blocks containing *WRKY* gene pairs, revealing strong conservation between the two legume species (*A. membranaceus* and *G. max*) and weaker synteny with the distantly related *A. thaliana*

The results of phylogenetic analysis indicated that the 94 members of the *AmWRKY* family and the 72 members of the *ArWRKY* family in *Arabidopsis thaliana* showed high similarity in diversity characteristics, but the distribution among subfamilies was significantly heterogeneous (Fig. 2A). Based on the evolutionary topology and sequence conservation, these WRKY family members were further classified into six evolutionary subfamilies, named Group I to Group VI (Table S5). Group I was the largest subfamily, containing 42 members (25 *AmWRKY* and 17 *ArWRKY* genes), suggesting that this subfamily might have undergone a gene expansion event, while Group V had the fewest members with only 3 genes. It is notable that the distribution ratios of different subfamilies in the WRKY families of the two plants were highly consistent, indicating that the core functions of the WRKY gene family remained highly conserved after species differentiation. However, the low abundance of Group V might reflect a reduced dependence of *A. membranaceus* on the functions of this subfamily during specific environmental adaptation processes, or there might be unannotated paralogous genes. Collinearity analysis indicated that more homologous WRKY gene pairs were identified between *Glycine max and Arabidopsis thaliana* (Fig. 2B). The collinearity of WRKY genes between *A. membranaceus* and *Glycine max* was significant, reflecting the conservation of legume genome evolution, while the collinearity with *Arabidopsis thaliana* was weak, demonstrating the differentiation of genome structure in distantly related species. This result provides important clues for understanding the evolution and functional differentiation of the WRKY gene family.

### Analysis of *AmWRKY* gene structure and motif

**Fig. 3:**
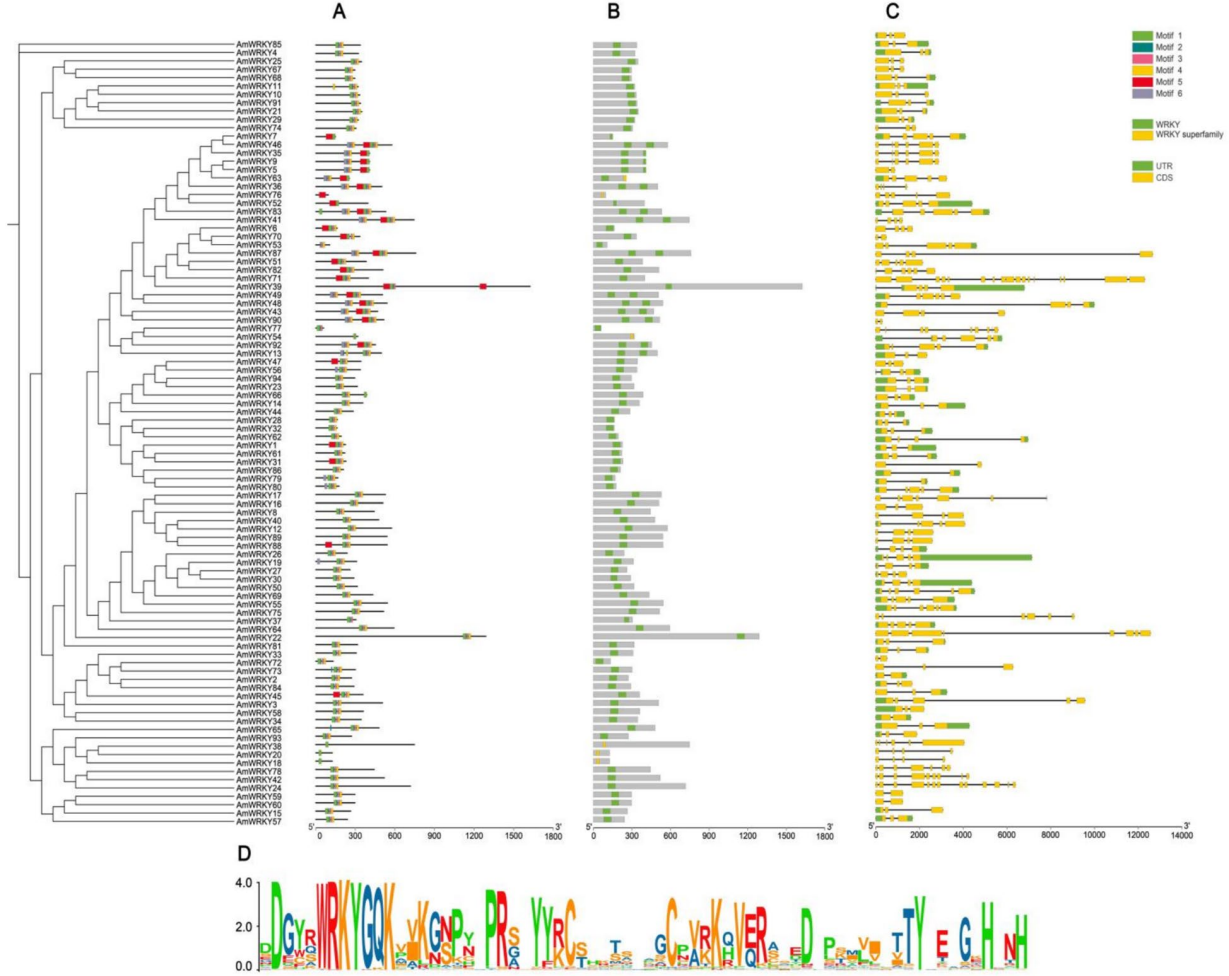
Gene structure and cis-element prediction of *AmWRKY* proteins. **A** Motif analysis of AmWRKY proteins. Different numbers represent different motifs. **B** Conserved domain and location information for each *AmWRKY* gene. **C** Structure of *AmWRKY* genes. A green rectangle indicates an untranslated region; a yellow rectangle indicates the coding sequence region; lines indicate introns. **D** Amino acid sequence information for motif 1. The x-axis of (**A**, **B**) is the number of amino acids. The X-axis of (**B**, **D**) is the number of nucleotides

The phylogenetic tree analysis results show that *AmWRKY* can be divided into multiple evolutionary subfamilies, and the topological structure of its branches shows significant consistency with the distribution of conserved motifs (Fig. 3A, B). Through the MEME online tool analysis, six motifs were identified with amino acid lengths ranging from 8 to 50, and each protein sequence contains at least 1 to 6 motifs. Using the Batch CD-Search online tool to identify the domains of all *AmWRKY* proteins, the results show that all members contain the characteristic domain of the WRKY gene family (Fig. 3C). In addition, the visualization analysis of the UTR, CDS and intron distribution characteristics of *AmWRKY* shows that the genes within the same phylogenetic branch have a highly similar number of introns, suggesting that the gene structure within each subfamily is highly conserved. Through Batch CD-Search to verify the domains of *AmWRKY* proteins, it was found that all members possess the core conserved domain “WRKYGQK” (Fig. 3D). This domain appears as a continuously highly conserved region in sequence alignment, thereby confirming the functional basis of DNA-binding activity for this protein family. The results indicate that *AmWRKY* has maintained high functional and structural conservation during long-term evolution, while the dynamic evolution of variable regions has driven functional diversity. These findings provide candidate targets for subsequent functional verification and molecular breeding for stress resistance.

### Analysis of cis-acting elements in *AmWRKY*

**Fig. 4:**
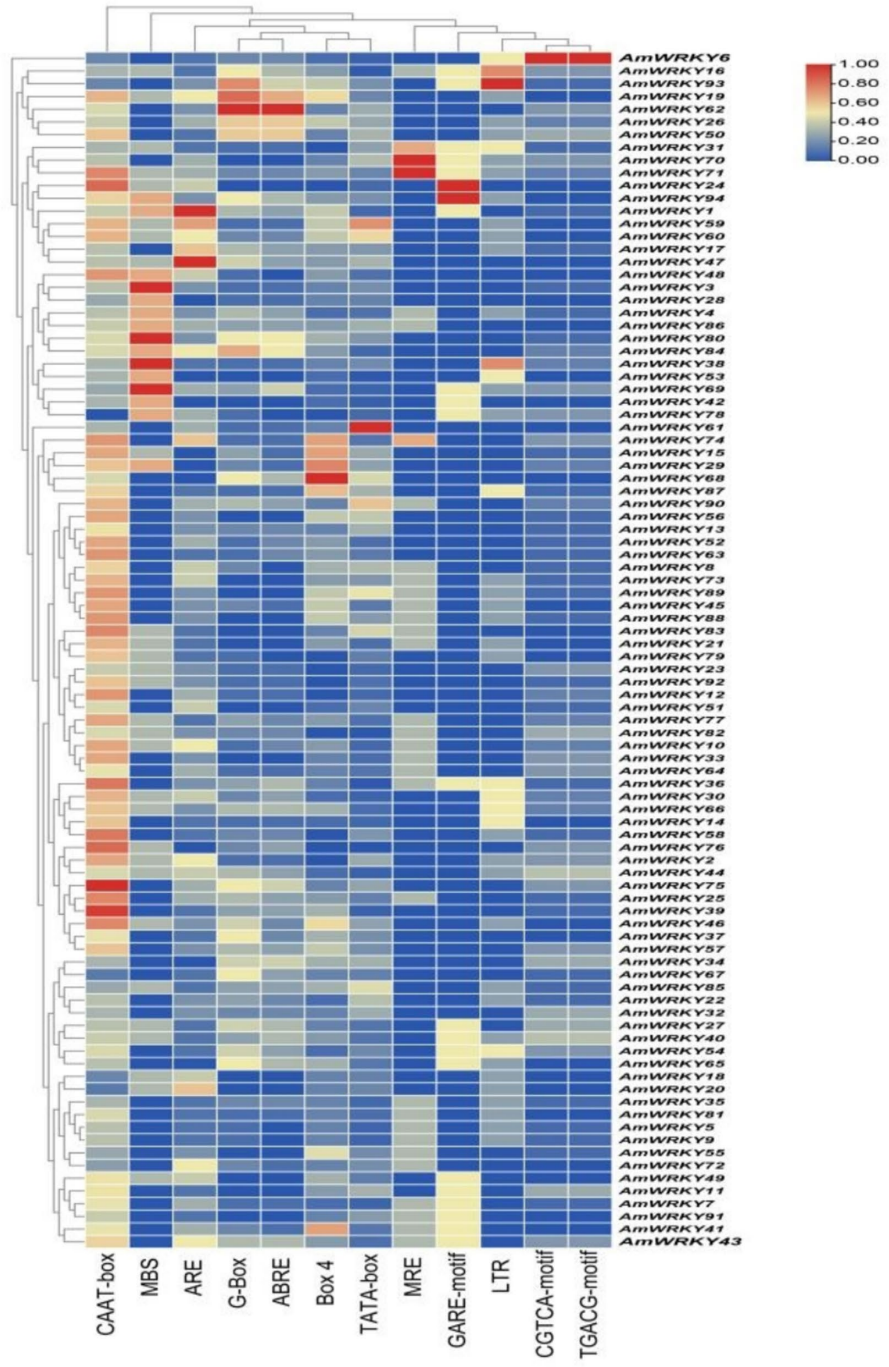
Statistical analysis of cis-elements in the promoter of *AmWRKY* gene. The color and number of the grid represent the number of cis-elements in corresponding *AmWRKY*

A systematic analysis of cis-regulatory elements in the promoter region was conducted. By extracting the 2000 bp promoter sequence upstream of the gene and performing functional annotation based on the PlantCARE database, three key regulatory modules were identified (Fig. 4). The first one is the growth and development regulatory elements, including the basic promoter elements TATA-box and CAAT-box. And the second one is the light response element G-Box and its conserved module Box 4, suggests that *AmWRKY* may be involved in the biological process of photoperiod regulation. The third one is the abiotic stress response elements, including the cold response element LTR, the oxidative stress element ARE, the drought induction element MBS (MYB binding site), and the metal ion sensing element MRE. It is worth noting that the MBS element shows a high frequency of distribution in the promoter region (frequency ≥ 5), suggesting that MYB transcription factors may mediate cold stress responses through this site. The plant hormone response elements, including the gibberellin response element GARE-motif, the jasmonic acid signaling element CGTCA/TGACG dual module, and the abscisic acid response element (ABRE) were also observed. These elements are highly consistent with the known functions of *AmWRKY* transcription factors in hormone signal transduction (Table S6).

### Expression analysis of *AmWRKY* under cold stress and qRT-PCR validation

**Fig. 5:**
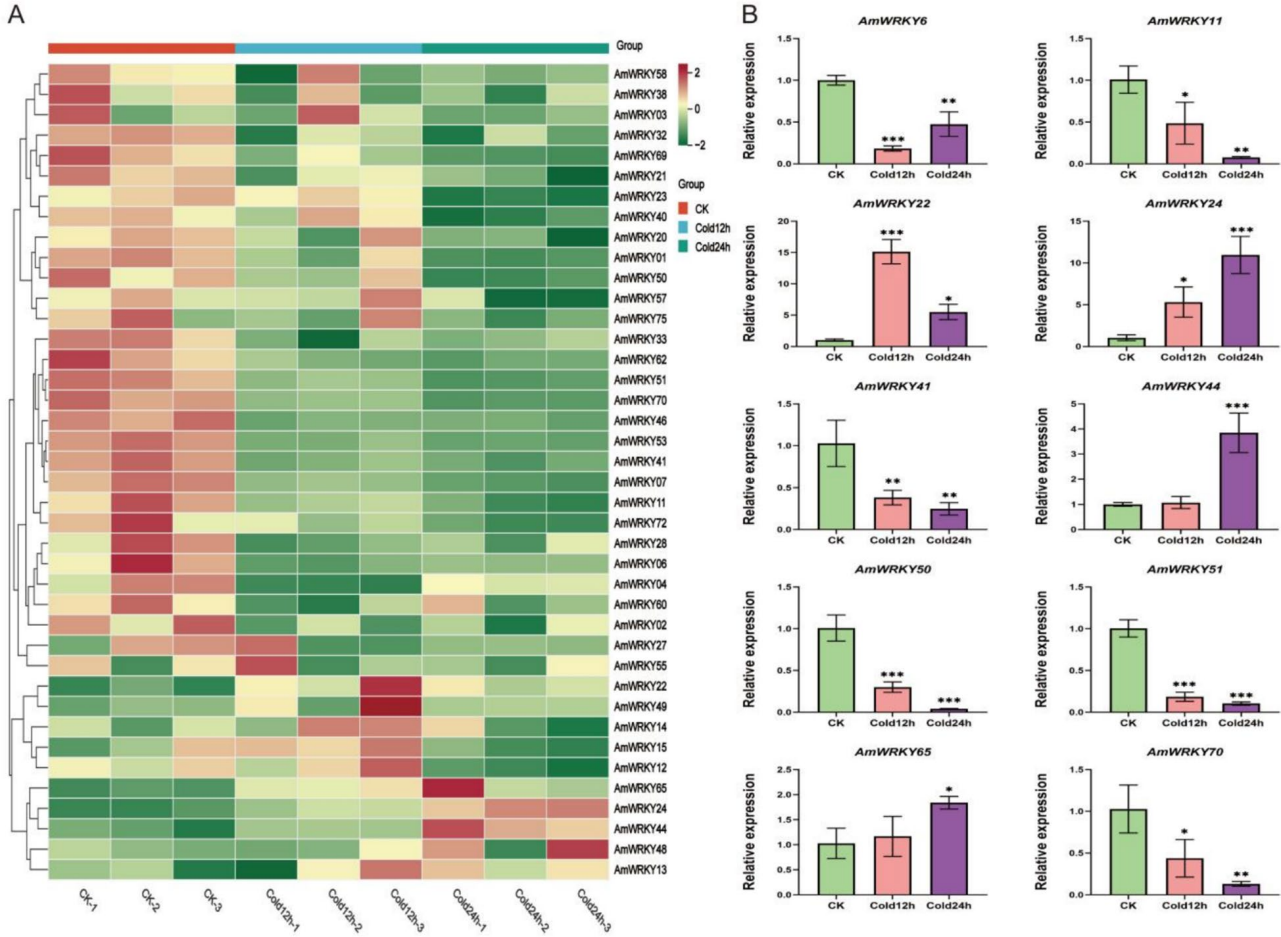
Heatmap of *AmWRKY* gene expression in *A. membranaceus* leaves under cold stress for different durations and qRT-PCR verification. **A** Heatmap of *AmWRKY* gene expression under cold stress. The FPKM values were processed using rowscaling transformation. Different colors indicate the degree of gene expression: red indicates higher expression and green lower expression. **B** Relative expression levels of 10 differentially expressed *AmWRKY* genes under cold stress for different durations detected by qRT-PCR. The X-axis indicates cold stress duration, the Y-axis indicates relative gene expression level. Error bars indicate the standard deviation of three repetitions. Asterisks denote a statistically significant difference (*p < 0.05, **p < 0.01, ***p < 0.001; Student’s t-test)

Gene expression pattern analysis is an important basis for understanding gene function. To investigate the regulatory mechanism of *AmWRKY* under cold stress, the expression characteristics of 94 *AmWRKY* genes using transcriptome data under cold stress were systematically analyzed (Fig. 5A). The data indicated that 40 *AmWRKY* genes exhibited a significant response to cold stress (FPKM value > 0.5; Table S7). Ten DEGs were identified in Cold12h vs CK. *AmWRKY22* and *AmWRKY24* were markedly upregulated, while the other 8 genes were downregulated. When the treatment duration was extended to 24 h, the number of DEGs increased to 16 (4 genes were upregulated and 12 were downregulated). Compared with the Cold12h group, six additional genes, namely *AmWRKY11*, *AmWRKY23*, *AmWRKY33*, *AmWRKY44*, *AmWRKY50*, and *AmWRKY65*, were detected. *AmWRKY22* and *AmWRKY24* remained continuously and significantly upregulated throughout the entire treatment period. In contrast, *AmWRKY44* and *AmWRKY65* were specifically highly expressed only in Cold24h. These findings suggest that these upregulated genes may play a positive role in regulating *A. membranaceus* cold resistance. Further analysis demonstrated that as the stress duration increased, both the number of DEGs and the extent of expression changes increased concurrently. This indicates that the cold response of this gene family exhibits remarkable time-dependent characteristics.

To validate the reliability of the transcriptome data, this study employed qRT-PCR to analyze the relative expression levels of 10 *AmWRKY* genes that showed significant differential expression. The results showed that the gene expression dynamics of Cold12h and Cold24h were highly consistent with the sequencing data (Fig. 5B), especially for key genes such as *AmWRKY22* and *AmWRKY24*, which remained significantly upregulated relative to the control levels throughout the entire treatment period, with *AmWRKY22* showing a peak response at Cold12h in both methods.This result confirmed the reliability of the transcriptome data, and also supported the prediction that *AmWRKY* has time-specific regulatory functions under cold stress through multi-time point validation.

### Construction and enrichment analysis of *AmWRKY* co-expression network

**Fig. 6:**
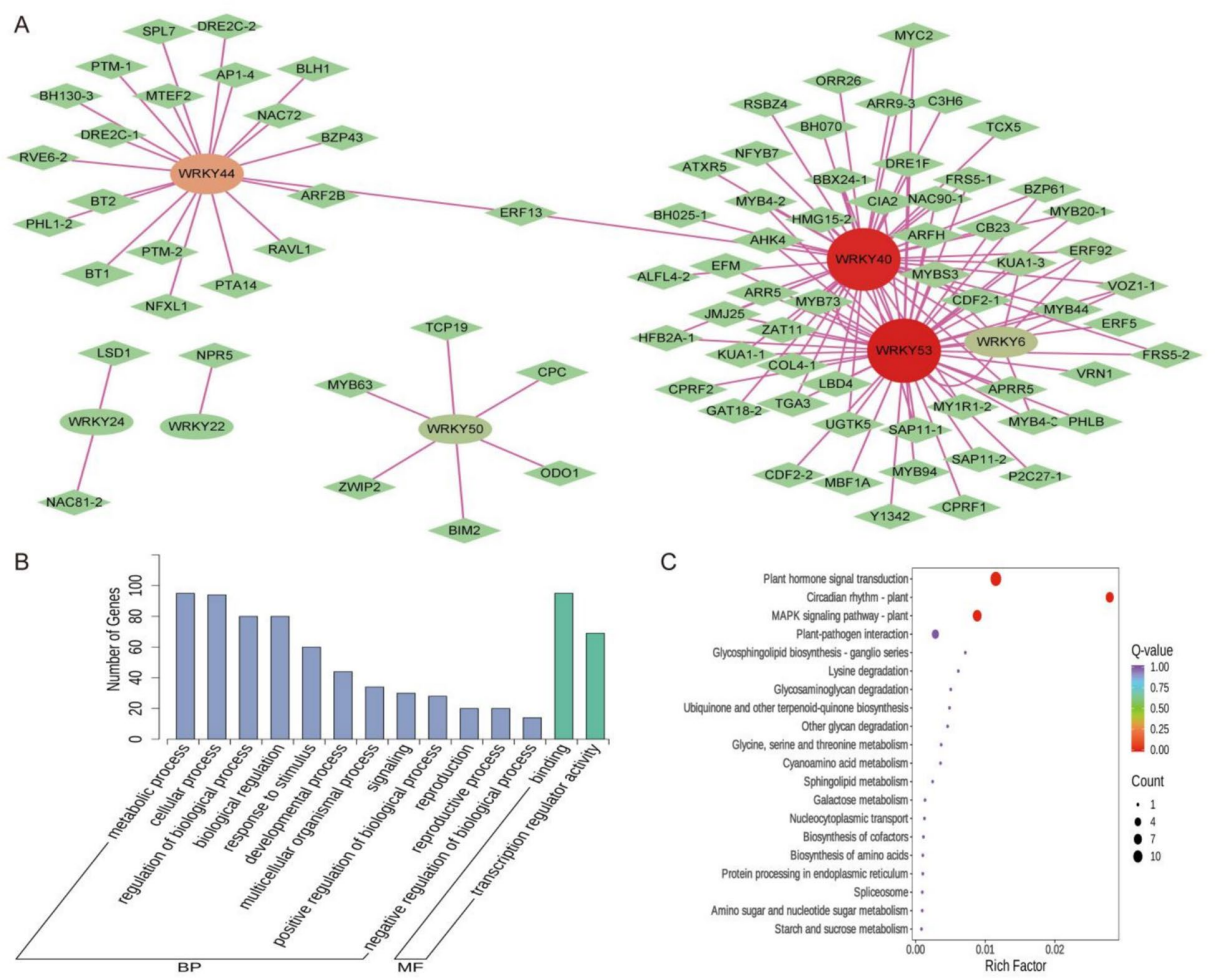
*AmWRKY* gene co-expression network constructed, and GO term and KEGG pathway enrichment analysis. **A** Co-expression network of *AmWRKY* genes constructed based on the transcriptome under salt stress. Diamonds indicate the *AmWRKY* genes and circles indicate other transcription factors. The color shade indicates the number of relevant genes. **B** GO term enrichment analysis of *AmWRKY* genes and co-expressed transcription factors. **C** KEGG pathway enrichment analysis of *AmWRKY* genes and co-expressed transcription factors

To elucidate the regulatory mechanism of *AmWRKY* under cold stress, a systematic analysis was conducted based on transcriptome data. A total of 3148 TFs from 91 gene families were identified. The bHLH (176 members), AP2/ERF—ERF (161 members), and MYB—related (135 members) gene families exhibited the highest abundances (Supplementary Fig. 1A). Python script was employed to screen for co-expressed genes, 89 candidate TFs from 44 gene families were found to co-express with 7 *AmWRKY* genes (FPKM > 0.5 and Pearson correlation coefficient *r* > 0.9). The AP2/ERF-ERF, MYB, and MYB-related families each contributed 8 members. These families have been well-documented to play extensive roles in plant responses to adverse environmental conditions (Fig. 6A; Table S8). This implies that *AmWRKY* may synergistically mediate cold response by forming a composite regulatory network with the aforementioned TFs.

A systematic analysis of the 7 *AmWRKY* and 89 TFs within the core network was conducted through Gene Ontology (GO) functional annotation. At the biological process level. These genes were predominantly involved in pathways such as metabolic process, cellular process, regulation of biological process, biological regulation, and growth. Regarding molecular functions, binding, transcription regulator activity, molecular transducer activity, and catalytic activity were the main categories. In terms of cellular components, they were significantly enriched in protein-containing complex (Supplementary Fig. 1B). Furthermore, GO and Kyoto Encyclopedia of Genes and Genomes (KEGG) enrichment analyses were performed. The results indicated that these genes were significantly enriched in molecular processes including DNA-templated transcription (90), RNA biosynthetic process (90), heterocycle biosynthetic process (90), DNA binding (76), regulation of primary metabolic process (69), regulation of metabolic process (69), regulation of nitrogen compound metabolic process (68), and regulation of biosynthetic process (67) (Fig. 6B). Additionally, they were highly enriched in key pathways, including hormone signaling (e.g., Plant hormone signal transduction), environmental adaptation (e.g., MAPK signaling pathway-plant), circadian rhythm, and plant-pathogen interaction (Fig. 6C). These findings reveal that *AmWRKY* may plays a central regulatory role in *A. membranaceus* under cold stress by establishing a coordinated regulatory network incorporating key transcription factors such as AP2/ERF and MYB. This network which integrates multiple pathways including hormone signaling, environmental perception, and metabolic regulation forms a crucial molecular foundation for *A. membranaceus* under cold stress.

### In-silico analysis of AmWRKY-mediated metabolic regulation under cold stress

**Fig. 7:**
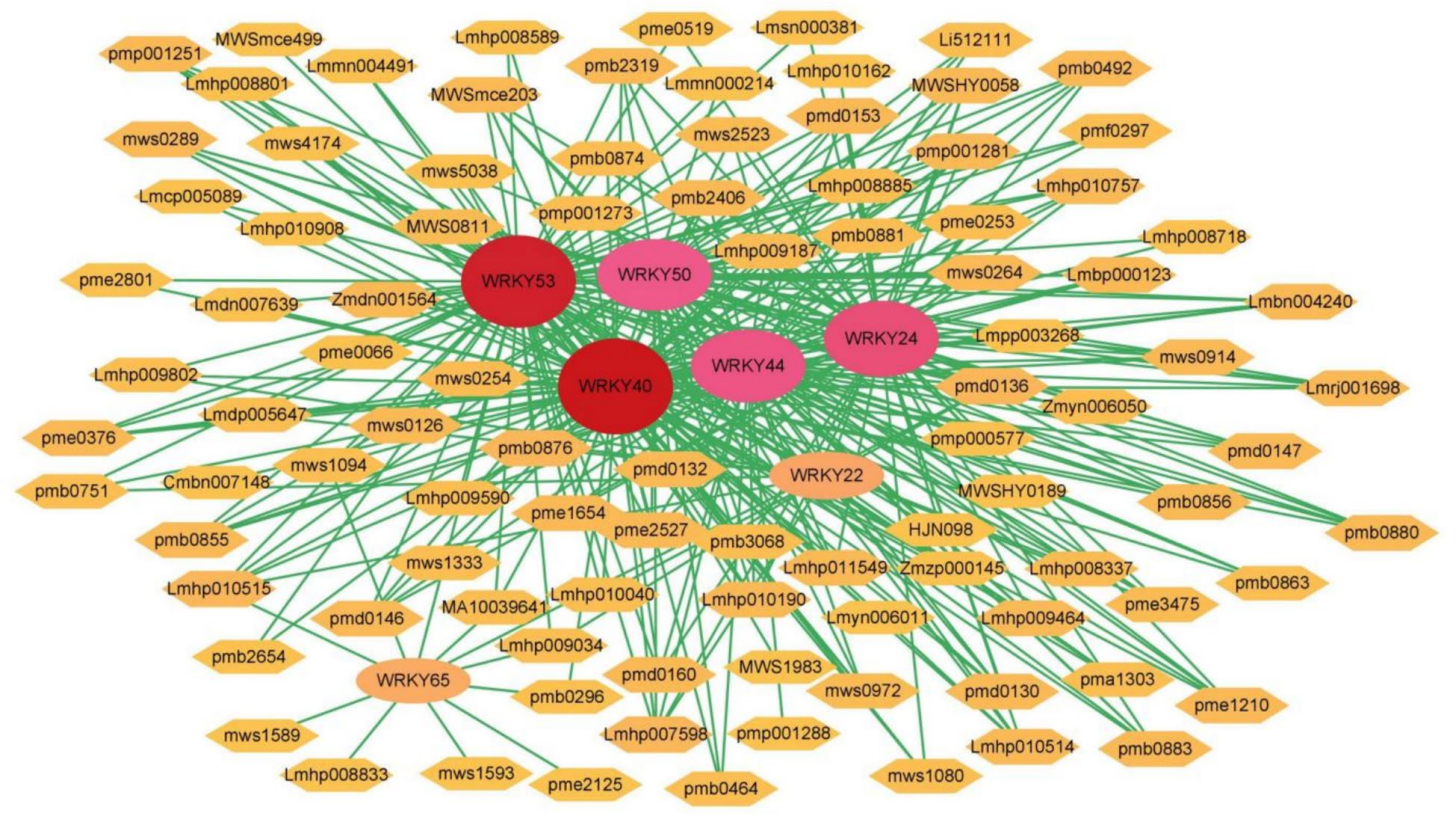
Pearson correlation network revealing the regulation by *AmWRKY* genes of metabolite synthesis of Am under cold stress. Circles indicate *AmWRKY* genes and diamonds indicate metabolites. The color shade indicates the number of genes associated with metabolites

This study systematically analyzed the metabolic regulatory mechanism of *AmWRKY* under cold stress by integrating transcriptome and metabolome data. DEGs were identified with criteria of |log₂ fold change (FC)|≥ 1.5 and false discovery rate (FDR) < 0.05. These were then subjected to a combined analysis with metabolites under cold stress. For metabolites that were significantly upregulated (log₂ FC ≥ 1.5, *p* < 0.05), metabolite-gene pairs with significant correlations were screened using the Pearson correlation coefficient (PCC, |r|> 0.8, *p* < 0.05). The research findings indicated that a total of 7 *AmWRKY* genes were significantly correlated with 98 metabolites. Among these, *AmWRKY22*, *AmWRKY24*, *AmWRKY44*, and *AmWRKY65* were significantly upregulated, whereas *AmWRKY40*, *AmWRKY50*, and *AmWRKY53* were significantly downregulated. The significantly upregulated metabolites predominantly consisted of 47 lipid-related compounds, 15 sugars, and 11 flavonoids (Fig. 7; Table S9). This suggests that *AmWRKY* may play a role in coping with cold stress by regulating the content alterations of lipids, sugars, and flavonoids in *A. membranaceus*.

**Fig. 8:**
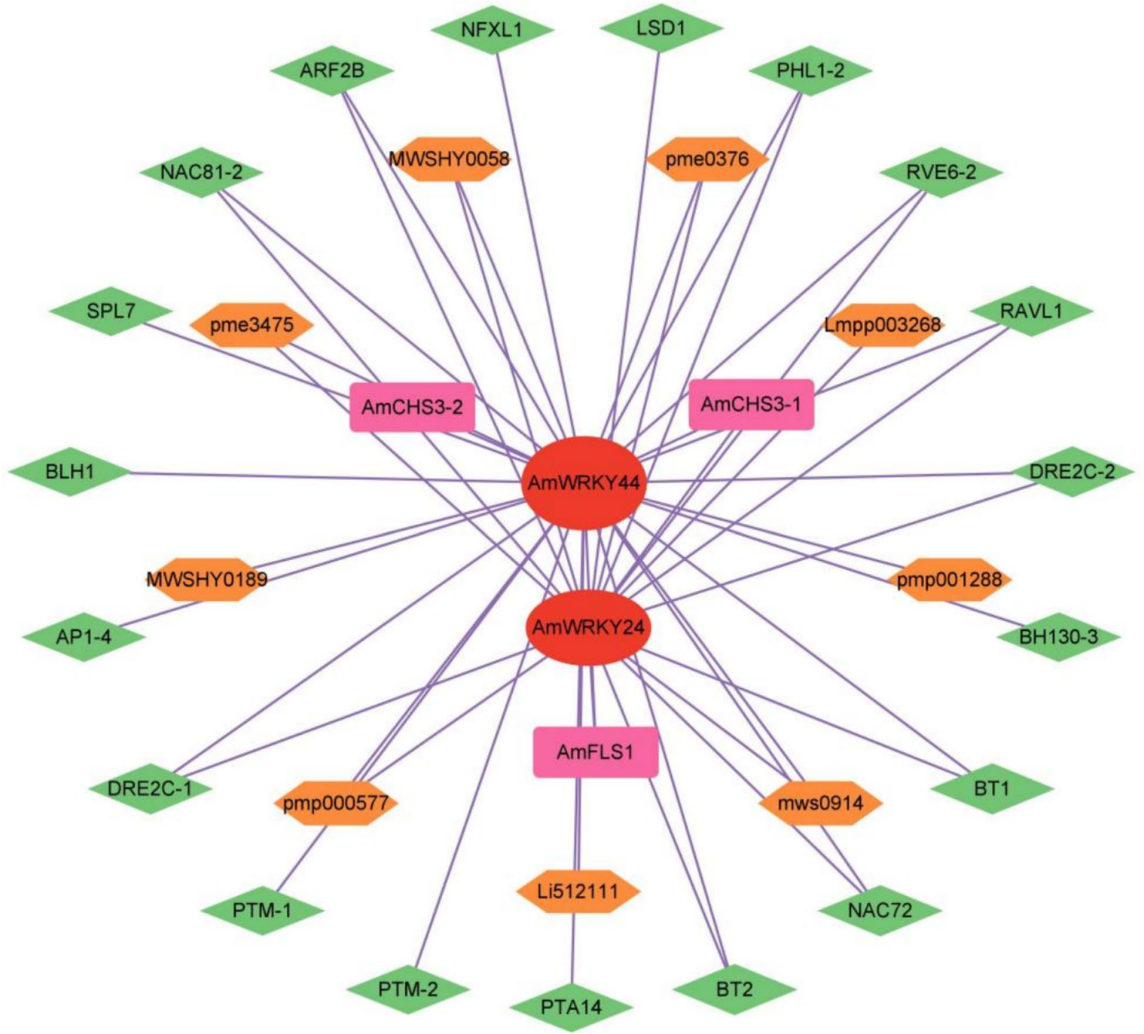
The Pearson correlation network revealed the regulation of *AmWRKY* gene and other TFs on flavonoid metabolite synthesis under cold stress. Different shapes of nodes present *AmWRKY* genes (circle), TF genes (diamond), functional gene (Rectangle) and metabolites (hexagon). Size of each node indicates quantities of correlated genes. The depth of the color indicates the degree of correlation

Flavonoids are not only the key indicators of the medicinal quality of *A. membranaceus*, but also important components in response to cold stress. Further investigations were conducted into the regulatory role of *AmWRKY* in the flavonoid metabolic pathway. A total of 17 functional genes within the flavonoid metabolic pathway were identified as being significantly upregulated under cold stress (Table S10), and 11 flavonoid metabolites were also found to be significantly upregulated (Table S11). By performing a correlation analysis of the co-expression network involving other transcription factors, it was discovered that 19 transcription factors, in concert with *AmWRKY24* and *AmWRKY44*, co-regulated the *AmCHS* and *AmFLS* genes. This co-expression pattern suggests a potential regulatory mechanism that coincides with an increase in the content of 9 flavonoid substances (Fig. 8). Based on these findings, it is conjectured that in *A. membranaceus*, there may exist a regulatory pathway of *AmWRKY24/44*-AmCHS/AmFLS-Flavonoids- enhancement of cold tolerance.

## Discussion

*A. membranaceus* is a valuable medicinal herb whose dried roots are rich in bioactive compounds like astragaloside IV, polysaccharides, and flavonoids, exhibiting multiple pharmacological effects [23, 24]. Driven by growing global demand for natural medicines, market needs have continuously risen [25]. However, over-harvesting has drastically reduced wild resources, with 90% of market supply now relying on cultivation [26]. Furthermore, climate-induced temperature fluctuations have altered the ecological environment of suitable growing areas, leading to unstable metabolite synthesis and significant batch-to-batch variation in medicinal quality [27]. Therefore, elucidating the molecular regulatory networks controlling growth and stress adaptation, particularly the roles of key transcription factors like WRKYs has become crucial for improving both yield and quality stability through molecular breeding.

As one of the largest and most functionally diverse transcription factor families [28], WRKY plays a pivotal role in regulating critical biological processes, including plant growth and development, responses to biotic and abiotic stresses, and hormone signal transduction [29, 30]. Members of this family contribute to plant stress adaptation by modulating hormone signaling pathways [31], also directly influence physiological processes such as cell wall biosynthesis, cellulose deposition regulation, and seed development, thereby playing an essential role in plant adaptive evolution [32]. In this study, 94 *AmWRKYs* were identified using bioinformatics methods, which is similar to the number of WRKY genes reported in most plants. The number of *AmWRKYs* (94) is significantly higher than that in *Arabidopsis thaliana* (72) and rice (28), but still relatively conservative compared to the legume-related species soybean (182), indicating that the WRKY gene family is widely present in different plants and plays an important role in plant growth and development as well as in response to environmental stress [33, 34].

The analysis of cis-acting elements in the promoters of all significantly upregulated *AmWRKY* genes revealed significant differences in the distribution of functional elements among different genes, which may be related to their specific functions in plant physiological processes. All genes contained the core CAAT-box and TATA-box promoter elements. *AmWRKY22* possessed significantly more TATA-boxes (80), suggesting strong basal transcriptional activity, while *AmWRKY24* had the highest number of CAAT-boxes (24), indicating greater promoter complexity. Additionally, *AmWRKY22*, *AmWRKY24*, and *AmWRKY44* contained 2, 4, and 4 antioxidant stress response elements (ARE), respectively, consistent with known roles in oxidative stress response such as those documented for *WRKY22* in Arabidopsis. This suggests that this gene plays a critical role in the regulation of reactive oxygen species (ROS) metabolism [35]. *WRKY24* and *WRKY44* in rice have been confirmed to significantly enhance the plants tolerance under adverse stress conditions by regulating the activity of antioxidant enzymes and the expression of related genes [36]. The promoter regions of *AmWRKY24*, *AmWRKY44*, and *AmWRKY65* each contained one low-temperature response element (LTR), suggesting their possible involvement in cold adaptability regulation.

The WRKY gene family originated in eukaryotes and expanded through fragment and tandem duplication events during plant evolution [37]. Cross-species collinearity is a key approach for studying genomic evolution and functional conservation. Numerous orthologous genes were identified between *A. membranaceus* and *Glycine max*, reflecting high gene conservation due to shared ancestry [38]. In contrast, far fewer orthologs were detected with *Arabidopsis thaliana*, suggesting greater genomic rearrangement or functional divergence. Phylogenetic analysis classified *AmWRKY* into six subfamilies. The notable expansion of Group I (42 members) may relate to its key role under cold stress. Notably, Group V contains only three members (*AmWRKY49*, *AmWRKY92*, and *AtWRKY71*), indicating possible reduced functional importance in environmental adaptation in *A. membranaceus*. *AtWRKY71* is a multifunctional regulator in Arabidopsis [39, 40], and based on orthology, *AmWRKY49* and *AmWRKY92* may have similar roles (Fig. 2A). Gene structure analysis showed high conservation within subfamilies, while variation in Motif 1–6 combinations may underlie functional divergence. These results provide new insights into WRKY evolutionary functional relationships in legumes.

*AmWRKY24* and *AmWRKY44* may enhance the plants adaptability to cold and drought dual stress by integrating LTR and MBS-mediated signals to coordinate the regulation of ABA signaling pathways and membrane stability. In contrast, *AmWRKY65*, lacking the MBS element, may rely more specifically on LTR to regulate the expression of cold response-related genes. Such cross-regulation of cold and drought signals at the molecular level has been reported in multiple species and homologous genes [41, 42]. Regarding plant hormone responses, *AmWRKY22*, *AmWRKY44*, and *AmWRKY65* contained 2, 3, and 4 ABRE, respectively. ABRE elements can bind to abscisic acid receptors, thereby regulating the expression of downstream genes and influencing the plant adaptability to drought, salt stress, and other adverse conditions [43]. Therefore, the higher number of ABRE indicates that they may play important roles in abscisic acid signaling. Especially *AmWRKY44*, which contains the most ABRE elements and may play a leading role in abscisic acid-mediated stress responses. The promoter region of *AmWRKY44* contained five copies of each of the two jasmonic acid (JA) signaling elements (CGTCA/TGACG), significantly higher than other family members, suggesting that it may play a core role in JA-mediated defense or developmental regulation (Table S6). This feature is similar to the function of *GhWRKY44* in cotton, which enhances plant resistance to pathogens by activating JA signal-related genes [44]. *AmWRKY24* and *AmWRKY65* contain 2 and 1 gibberellin response elements (GARE), suggesting that they may be involved in gibberellin-related physiological processes. Similarly, *ZmWRKY65* in maize enhances the stress resistance of transgenic *Arabidopsis* by regulating the expression of gibberellin pathway genes [45], and *TaWRKY24* in wheat enhances drought resistance by up-regulating the expression of dehydroascorbate reductase (DHAR) and interacting with TaERFL1a [46]. All these results indicating that WRKY may coordinate plant growth and environmental adaptability through the gibberellin signaling pathway.

The mechanism of WRKY transcription factors in plant response to abiotic stress is complex and diverse. They improve plant stress tolerance by directly modulating the expression of downstream genes or by establishing intricate regulatory networks via interactions with other TFs [47]. Through systematic transcriptomic profiling under cold stress in *A. membranaceus*, we identified co-expression networks (Pearson’s r > 0.9, *p* < 0.001) bridging of *AmWRKY* and key regulators from the AP2/ERF, MYB, and MYB-related families. This synergistic interplay implies their collective role in orchestrating cross-pathway signal integration, potentially through cis-element co-binding or hierarchical transcriptional cascades. Evolutionary conservation of this regulatory paradigm is evidenced by analogous cooperation among AP2/ERF, MYB, and WRKY homologs in numerous plant species during salicylic acid-mediated stress adaptation [48, 49]. These TFs also showed high co-expression in regulating plant metabolic pathways and signal transduction processes [50]. The study revealed that the AP2/ERF-ERF, MYB, and MYB-related families each contributed 8 members that were co-expressed with *AmWRKY*. These findings imply that these TFs might form a composite regulatory module within *A. membranaceus*. They were associated with crucial biological pathways such as plant hormone signal transduction, the MAPK signaling pathway, and circadian rhythms. MPK3 and MPK6, as pivotal kinases, are capable of phosphorylating the WRKY33 protein in *Arabidopsis thaliana* [51]. This phosphorylation enhances the protein DNA-binding activity and transcriptional activation ability.

The distribution of light signal response elements (G-Box) shows that *AmWRKY22*, *AmWRKY44*, and *AmWRKY65* contain 3, 4, and 6 G-Boxes, respectively, while *AmWRKY24* is completely lacking, suggesting that the former three may be regulated by light signals. The significant enrichment of the “circadian rhythm-plant” pathway within the core network (with 7 genes) suggests a potential temporal connection between the cold response and the regulation of the biological rhythms. Research has demonstrated that *ArWRKY40* can coordinate the circadian rhythm of stress responses by regulating the core components of the biological rhythms [52]. In this study, the co-expression of *AmWRKY* with genes related to the circadian rhythm may indicate that *A. membranaceus* enhances its environmental adaptability by dynamically regulating the expression of stress-resistance genes through biological rhythms.

WRKYs typically exert an influence on the synthesis and accumulation of plant metabolites by modulating the expression of downstream genes. These factors are capable of activating genes associated with sugar metabolism under cold stress, thereby enhancing the level of sugar metabolism to bolster the plant’s cold tolerance [53]. Additionally, they are involved in the biosynthesis of secondary metabolites such as lignin, phenolic compounds, and flavonoids, thereby playing a crucial role in cold resistance and protection [34]. Seven *AmWRKYs* were found to exhibit a significant correlation with 98 cold-responsive metabolites (|r|> 0.8, *p* < 0.05) based on the integration of transcriptomic and metabolomic data (Fig. 7). Among these, *AmWRKY22*, *AmWRKY24*, *AmWRKY44*, and *AmWRKY65* were significantly upregulated, whereas *AmWRKY40*, *AmWRKY50*, and *AmWRKY53* were significantly downregulated. The differentially abundant metabolites were predominantly enriched in lipid-related compounds (47 types), carbohydrates (15 types), and flavonoids (11 types) (Fig. 7, Table S8). Previous research has shown that WRKYs can modify lipid metabolism (such as membrane lipid unsaturation) by regulating genes related to membrane lipid degradation/oxidation, thereby maintaining the stability of cell membranes under cold stress [54, 55]. Moreover, they can promote the synthesis and transport of non-structural carbohydrates like sucrose, alleviating cold damage through osmotic protection and energy provision [56]. These findings suggest that *AmWRKY* may coordinate various mechanisms, including lipid metabolism, flavonoid synthesis, the accumulation of osmoregulatory substances, and the synthesis of antioxidants, to collaboratively maintain cellular homeostasis in *A. membranaceus* under cold stress. The significant cold-induction of 40 *AmWRKY* genes provides the molecular basis for *A. membranaceus*’s adaptation to cold habitats. Despite its phenotypic sensitivity, this extensive *AmWRKY* response reveals a robust genetic defense mechanism. The sustained upregulation of key members like “*AmWRKY22/24/44/65*” suggests they orchestrate the adaptive physiological and metabolic changes necessary for survival in its high-altitude, cold native environment.

Flavonoids are not only the core indicators of the medicinal active components of *A. membranaceus* but also the key metabolic products for plants to resist cold stress. Flavonoids in *A. membranaceus* exhibit a notable accumulation under cold stress, and this accumulation is intricately linked to the plant's cold tolerance capacity [57]. Moreover, flavonoids enhance the overall stress resistance of *A. membranaceus* by modulating its secondary metabolic pathways [58]. This study further uncovers the central roles of *AmWRKY24* and *AmWRKY44* within the regulatory network of flavonoid biosynthesis. It was found that *AmWRKY24/44* forms a synergistic regulatory module in conjunction with 19 TFs under cold stress, including NAC, MYB, and bHLH through the construction of a transcriptional regulatory network. This module regulates the key genes *AmCHS* and *AmFLS* involved in flavonoid synthesis (Fig. 8), leading to a significant augmentation in the accumulation levels of nine flavonoids, such as luteolin (Table S9). This study provides strong in-silico evidence supporting the crucial role of WRKY transcription factors in plant secondary metabolism and, for the first time, proposes the regulatory pathway of *AmWRKY24/44*-target genes (*AmCHS/FLS*) leading to flavonoid-mediated cold resistance in *A. membranaceus*. *WRKY50* directly interacts with bHLH92, MYB19, and NAC44 to jointly govern the expression of genes responsive to cold stress in wheat [59]. These findings mutually corroborate the regulatory model proposed, indicating that *AmWRKY* may integrate cold environmental cues and collaborate with other TFs to regulate *A. membranaceus* flavonoid synthesis. Consequently, the regulatory model put forward in this study offers a novel perspective for elucidating the molecular mechanisms underlying plant cold-resistance traits, also lays a theoretical foundation for gene-editing strategies aimed at optimizing the flavonoid synthesis pathway. The proposed regulatory model, derived from co-expression and correlation networks, necessitates further functional validation. Future work involving the characterization of key *AmWRKY* genes through transgenic assays and promoter binding studies will be crucial to solidify the molecular mechanisms outlined in this work.

## Conclusion

This study systematically identified the *AmWRKY* gene family at the whole-genome level, and comprehensively analyzed their physicochemical properties, chromosomal localization and distribution, gene structure characteristics, evolutionary relationships, and expression patterns. By integrating transcriptomic and metabolomic data, key *AmWRKY* potentially involved in regulating the synthesis of secondary metabolites in *A. membranaceus* were mined, further proposing a potential regulatory model of *AmWRKY24/44* → flavonoid biosynthesis → enhanced cold adaptability. These results deepen the understanding of the mechanism of the *AmWRKY* gene family in regulating the growth and development and the synthesis of secondary metabolites, also provide important theoretical basis for screening key genes for genetic improvement to enhance the stress resistance in *A. membranaceus*. Future perspectives include experimental validation of the proposed AmWRKY24/44-flavonoid pathway through molecular assays and functional studies to advance molecular breeding for cold-resistant *A. membranaceus*.

## Supplementary Information

Supplementary Material 1: Fig. S1: Statistical analysis of transcription factors based on *A. membranaceus* transcriptome data under cold stress and GO term annotations of AmWRKY genes and co-expressed transcription factors. (A) Statistical analysis of transcription factors based on *A. membranaceus* transcriptome data under cold stress; (B) GO term annotations of AmWRKY genes and co-expressed transcription factors. BP, biological processes; CC, cellular components; MF, molecular functions.

Supplementary Material 2: Supplementary Tables. Table S1: The qPCR primers sequences used in this study. Table S2: The list of 94 WRKY genes identified in this article. Table S3: The subcellular localization prediction results of 94 AmWRKY genes. Table S4: List of Ka/Ks analysis for AmWRKY duplicated gene pairs. Table S5: Phylogenetic analysis of AmWRKY protein. Table S6: The list of cis-elements of AmWRKY genes promoter. Table S7: Expression data of AmWRKY genes in *A. membranaceus* leaf under different times cold stress (CK:0h, Cold12h:12h, Cold24h:24h). Table S8: List of transcription factors co-expressed with AmWRKY genes. Table S9: List of 98 significantly up-regulated metabolites in *A. membranaceus* under cold stress. Table S10: List of Flavonoid Metabolite Information Significantly Upregulated in *A. membranaceus* under cold Stress. Table S11: List of Significantly Upregulated Functional Genes in the *A. membranaceus* Flavonoid Metabolic Pathway under cold Stress.

## Data Availability

All data generated or analyzed during this study are included in this article and its supplementary information files. All materials are available through corresponding authors upon reasonable request.
