# Supplementary figures and images for: Genome-wide identification of the WRKY transcription factors family and regulation of metabolites under cold stress in Astragalus membranaceus
Source: BMC Plant Biol. 2025 Nov 28;25:1663. doi: 10.1186/s12870-025-07685-2 (PMC12664180; doi:10.1186/s12870-025-07685-2)

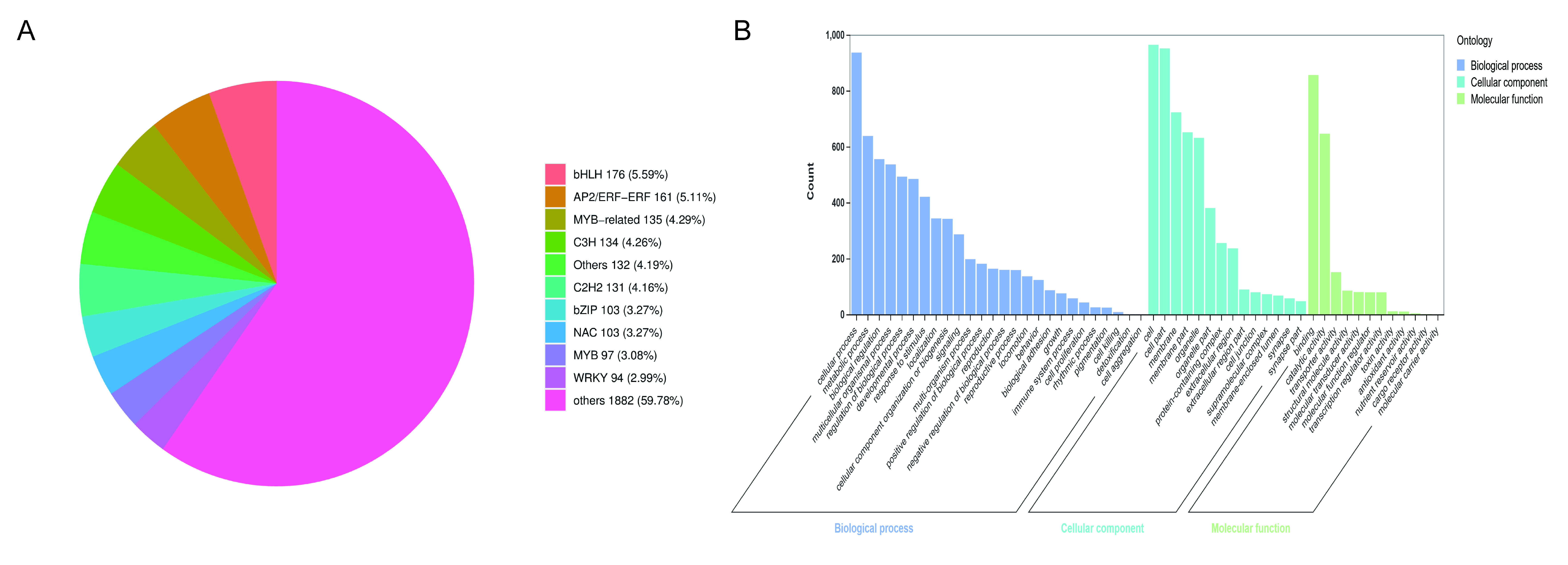

Supplement: Supplementary file 1 — Supplementary Material 1: Fig. S1: Statistical analysis of transcription factors based on A. membranaceus transcriptome data under cold stress and GO term annotations of AmWRKY genes and co-expressed transcription factors. (A) Statistical analysis of transcription factors based on A. membranaceus transcriptome data under cold stress; (B) GO term annotations of AmWRKY genes and co-expressed transcription factors. BP, biological processes; CC, cellular components; MF, molecular functions. [file 12870_2025_7685_MOESM1_ESM.tif]
